# Chromosomal profile of indigenous pig (*Sus scrofa*)

**DOI:** 10.14202/vetworld.2015.183-186

**Published:** 2015-02-16

**Authors:** P. Guru Vishnu, B. Punyakumari, B. Ekambaram, M. Gnana Prakash, B. V. Subramanyam

**Affiliations:** Department of Animal Genetics and Breeding, Sri Venkateswara Veterinary University, Tirupati, Andhra Pradesh, India

**Keywords:** chromosomal profile, indigenous pigs, idiogram, morphometric measurements

## Abstract

**Aim:**

The objective of this study was to investigate the chromosomal profile of indigenous pigs by computing morphometric measurements.

**Materials and Methods:**

A cytogenetic study was carried out in 60 indigenous pigs to analyze the chromosomal profile by employing the short term peripheral blood lymphocyte culture technique.

**Results:**

The modal chromosome number (2n) in indigenous pigs was found to be 38 and a fundamental number of 64 as in the exotic. First chromosome was the longest pair, and thirteenth pair was the second largest while Y-chromosome was the smallest in the karyotype of the pig. The mean relative length, arm ratio, centromeric indices and morphological indices of chromosomes varied from 1.99±0.01 to 11.23±0.09, 1.04±0.05 to 2.95±0.02, 0.51±0.14 to 0.75±0.09 and 2.08±0.07 to 8.08±0.15%, respectively in indigenous pigs. Sex had no significant effect (p>0.05) on all the morphometric measurements studied.

**Conclusion:**

The present study revealed that among autosomes first five pairs were sub metacentric, next two pairs were sub telocentric (6-7), subsequent five pairs were metacentric (8-12) and remaining six pairs were telocentric (13-18), while both allosomes were metacentric. The chromosomal number, morphology and various morphometric measurements of the chromosomes of the indigenous pigs were almost similar to those established breeds reported in the literature.

## Introduction

India, with an estimated population of 10.29 ­million pigs [1] accounts for 2.01% of the total livestock population and Andhra Pradesh ranks 9^th^ in the country with a pig population of 0.39 millions [1,2]. Pork production in the country stands at 0.459 million tons as against the world production level of 103.19 million tons [2]. The Per capita availability of meat in India is estimated to be 5 kg/year which is much lower than ICMR recommendation (10.8 kg). Hence, pig farming could play a vital role in augmenting the animal protein supply to the growing needs of emerging population.

Livestock biodiversity is critically important for achieving food security and alleviating poverty for the rapidly growing human population [3]. Among various animals, pig farming plays an important role in the livelihood of many families in the developing world. Pigs mostly found in the rural areas are variously referred to as native, scavenging, indigenous, and local or village pigs. Native pigs are generally hardy, survive and reproduce on a low plane of nutrition and require minimal inputs in terms of family labor. The scavenging pig has been rightfully or wrongly described as a dirty animal, an object of distaste and a parasite trafficker to humans. When kept under well-managed confined conditions, the pig is the exact opposite of the above description [4]. Local pigs have been documented by several authors as important assets because they improve livelihoods of rural farmers [5]. Indigenous pigs are well adapted to tropical conditions as they are adapted to local production conditions and environments [6]. They are also less susceptible to common diseases and parasites. In addition, the local breeds have the ability to survive long periods of feed and water deprivation [5] compared to exotic breeds.

Cytogenetics can be used to select reproducers free from chromosome abnormalities, which are responsible for abnormal body conformation (aneuploidy), lower fertility (balanced chromosome abnormalities) or sterility (sex chromosome abnormalities). Cytogenetics plays a significant role in identifying X-trisomy, Sex-reversal syndrome, XX/XY mosaicism and 1/29 Robetrsonian translocation, etc. [7]. For the evolution of species, cytogenetics has been recognized as an important tool, besides conventional classification and numerical taxonomy [7].

Cytogenetic studies have been widely carried out in all domestic animal species with little attention given to pigs in India. Therefore, goal of this study was to bridge this gap by investigating the characteristics of the indigenous pigs at the chromosomal level by computing morphometric measurements.

## Materials and Methods

### Ethical approval

Blood samples were collected aseptically with adequate precautionary measures to minimize pain and/or discomfort to the animals and also collection of samples was carried out in accordance with the guidelines laid down by the International Animal Ethics Committee and prevailing local laws and regulations.

### Study area

Tirupati is a major pilgrimage and cultural city in the Chittoor district of Andhra Pradesh. Tirupati is located about 20 km North West of Tirupati in the Tirumala hills at an elevation of 853 m. The present study was investigated in the Department of Animal Genetics and Breeding, College of Veterinary Science, Sri Venkateswara Veterinary University, Tirupati.

### Experimental animals

A chromosomal study was conducted among 60 indigenous pigs (30 boars and 30 sows) present in and around Tirupati maintained under scavenging system by weaker sections communities, which mainly act as both insurance to the rainfed agriculture and for meat consumption. Indigenous strains are mostly small to medium in size, hairy and black colored with an elongated face and short ears. They are potbellied, some of them are white striped on the neck or shoulders and spotted on the fore-head. They possess on average a litter size of 4-8 piglets, individually weigh about 0.5-0.8 kg at birth, 4.0-7.0 kg at weaning and 10-14 kg at 180 days. The experimental animals included in this study were selected randomly from group of animals maintained by weaker sections.

### Technique

Blood (2 ml/animal) was collected aseptically from external jugular vein and cultures were set up as per the short term lymphocyte culture method given by Moorehead *et al*. [8] with slight modifications. Initially each blood sample (0.5 ml) was cultured for 72 h. in 8 ml of RPMI 1640 tissue culture media supplemented with Phytohemagglutinin–M (0.1 ml), Fetal bovine serum (1.5 ml) and finally 40 µl colchicine (0.4 μg) was added 1½ h prior to harvesting to arrest cells at the metaphase stage followed by hypotonic treatment (0.075M KCl) for 30 min at 37°C and fixation in methanol: Glacial acetic acid (3:1). Air-dried slides were prepared and stained with 2% Giemsa for 20 min. Metaphase spreads with clear staining, and non-overlapping chromosomes were photographed (× 1000) and karyotyped.

### Statistical analysis

Data on Morphometric measurements such as relative length, arm ratio, centrometric index and morphological index was subjected to statistical analysis (One-way ANOVA) as per the standard procedure recommended by Snedecor and Cochran [9].

## Results and Discussion

**Figure-1:**
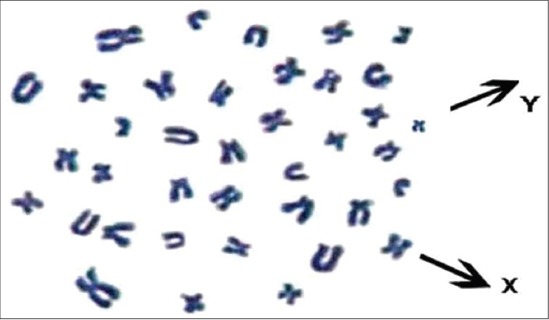
Giemsa stained metaphase spread of indigenous Boar (2n = 38, XY)

**Figure-2:**
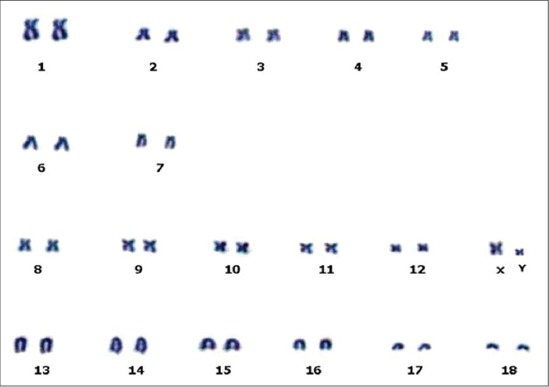
Giemsa stained Karyotype of indigenous Boar (2n = 38, XY)

**Figure-3:**
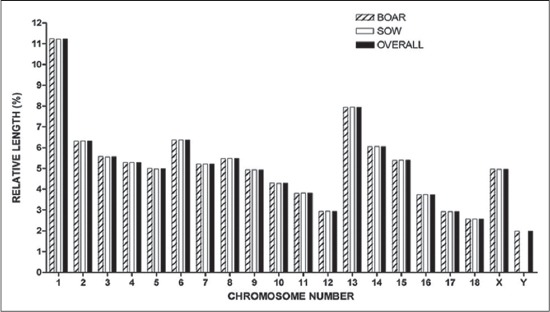
Idiogram showing relative length (%) of chromosomes of indigenous pig

The present study involved analysis of total 900 metaphase plates and revealed a modal diploid chromosome number (2n) of 38, which includes 18 pairs of autosomes and one pair of allosomes (Figure-1). Each pair was significantly (p<0.01) different from each other with respect to each morphometric measurement (Table-1). First 5 pairs of autosomes were submetacentric, next two pairs were subtelocentric (6-7), subsequent 5 pairs were metacentric (8-12) and remaining six pairs were telocentric (13-18) and allosomes were metacentric in nature (Figure-2). First chromosome was the longest pair and thirteenth pair was the second largest, while Y-chromosome was the smallest in the karyotype (Figure-3) of the pig, which was in agreement with the findings of earlier researchers [10-16].

**Table-1:** Morphometric measurements of chromosomes (mean±SE) in indigenous pigs.

Chromo-some no	Arm ratio				Centromeric indices				Morphological indices			
		
	Boar	Sow	Overall	Boar	Sow	Overall	Boar	Sow	Overall
1	1.89±0.04^a^	1.90±0.07^a^	1.89±0.17	0.65±0.04^a^	0.66±0.08^a^	0.65±0.08	8.09±0.17^a^	8.07±0.16^a^	8.08±0.15
2	2.03±0.06^b^	2.03±0.06^b^	2.03±0.06	0.67±0.00^b^	0.67±0.05^b^	0.67±0.00	4.45±0.06^b^	4.43±0.12^b^	4.44±0.04
3	1.87±0.04^c^	1.87±0.13^c^	1.87±0.01	0.65±0.01^c^	0.65±0.04^c^	0.65±0.03	4.31±0.01^c^	4.29±0.08^c^	4.30±0.08
4	1.76±0.05^d^	1.76±0.06^d^	1.76±0.00	0.64±0.01^d^	0.64±0.06^d^	0.64±0.04	4.27±0.00^d^	4.26±0.05^d^	4.27±0.06
5	1.41±0.04^e^	1.40±0.06^e^	1.40±0.04	0.58±0.00^e^	0.58±0.02^e^	0.58±0.07	4.62±0.04^e^	4.61±0.04^e^	4.62±0.11
6	2.96±0.02^f^	2.95±0.07^f^	2.95±0.02	0.75±0.01^f^	0.75±0.02^f^	0.75±0.09	3.18±0.02^f^	3.16±0.06^f^	3.17±0.09
7	2.92±0.03^g^	2.92±0.06^g^	2.92±0.03	0.74±0.00^g^	0.74±0.07^g^	0.74±0.10	2.52±0.03^g^	2.50±0.02^g^	2.51±0.13
8	1.26±0.03^h^	1.26±0.13^h^	1.26±0.08	0.56±0.00^h^	0.56±0.11^h^	0.56±0.05	5.94±0.08^h^	5.93±0.02^h^	5.94±0.01
9	1.24±0.03^i^	1.24±0.06^i^	1.24±0.03	0.55±0.00^i^	0.55±0.06^i^	0.55±0.09	5.32±0.03^i^	5.33±0.07^i^	5.32±0.08
10	1.11±0.04^j^	1.12±0.07^j^	1.11±0.04	0.53±0.01^j^	0.53±0.13^j^	0.53±0.17	5.15±0.04^j^	5.14±0.11^j^	5.15±0.04
11	1.08±0.03^k^	1.08±0.06^k^	1.08±0.03	0.52±0.01^k^	0.52±0.02^k^	0.52±0.13	4.14±0.03^k^	4.12±0.06^k^	4.13±0.06
12	1.04±0.04^l^	1.04±0.13^l^	1.04±0.05	0.51±0.01^l^	0.51±0.02^l^	0.51±0.14	3.47±0.05^l^	3.45±0.13^l^	3.46±0.02
X	1.18±0.05^m^	1.18±0.07^m^	1.18±0.08	0.54±0.01^m^	0.54±0.01^m^	0.54±0.01	5.71±0.08^m^	5.69±0.06^m^	5.70±0.02
Y	1.21±0.06	-	1.21±0.04	0.55±0.07	-	0.55±0.06	2.08±0.04	-	2.08±0.07

SE=Standard error, Means followed by different superscripts (column wise) differ significantly (p<0.01), Means followed by different superscripts (row wise) differ significantly (p<0.05)

### Relative length

The mean relative length of autosomes varied from 2.56±0.01 to 11.23±0.09%, while the relative contribution of X and Y chromosome was 4.96±0.07 and 1.99±0.01%, respectively, which were in ­accordance with the mean relative lengths as reported earlier in other breeds like Indian domestic pigs [17-20], Canadian breeding boar [21] and Nigerian Indigenous Pig [22]. Sex had no significant effect (p>0.05) on the relative length of all chromosomes, which revealed that the contribution of genetic information by autosomes and allosomes was similar irrespective of the sex of the animal. The idiogram showing the relative lengths of chromosomes was depicted in Figure-3.

### Arm ratio

The mean arm ratios of autosomes in indigenous pigs varied from 1.04±0.05 (12^th^ chromosome) to 2.95±0.02 (6^th^ chromosome), while the corresponding mean arm ratios of X and Y chromosomes were 1.18±0.08 and 1.21±0.04 (Table-1), which were within the range of the means reported in Indian domestic pigs [17-20] and Niang Megha Pig [11]. The first 12 chromosome pairs differed significantly from each other in arm ratios indicating that the proportion of the chromosome arm was different in the chromosomes. The effect of sex was not significant (p>0.05) on arm ratios of first 12 chromosomes, which revealed that morphology of biarmed autosomes was similar in both the sexes.

### Centromeric index

The mean centromeric indices estimated for the autosomes ranged from 0.51±0.14 to 0.75±0.09 (Table-1). The overall mean centromeric indices of X and Y chromosome was 0.54±0.01 and 0.55±0.06, respectively. The present findings were comparable with the results of Sahoo *et al*. [11] and Apparao *et al*. [17] in Indian domestic pigs. The influence of sex on centromeric indices of all the chromosomes was found to be non-significant (p>0.05) in the present study even though males possess higher centromeric index when compared with females.

### Morphological index

The overall mean of morphological indices for the autosomes and allosomes varied from 2.08±0.04 to 8.09±0.17% in males and 2.50±0.02 to 8.07±0.16% in females with an overall mean of 2.08±0.07-8.08±0.15, while X and Y chromosome had a morphological indices 5.70±0.02 and 2.08±0.07, respectively (Table-1), which corroborated with the findings of Sahoo *et al*. [11] and Guruvishnu *et al*. [12] in Indian domestic pigs. Since, morphological index is directly proportional to the total length (p+q) of chromosome, variation in total length results in variation in the relative length of chromosomes which in turn results in variation in morphological index. However, sex had no significant effect (p>0.05) on morphological index.

## Conclusion

The modal chromosome number (2n) in indigenous pigs was found to be 38 and a fundamental number of 64 as in the exotic. The present study concluded that the animals screened were free from chromosomal abnormalities and sex had no significant effect on all the morphometric measurements studied. This information will be of tremendous benefit in pig breeding program aiming at enhancing meat production, disease resistance and adaptation to environmental stress (climate change). The published literature for the effect of genetic groups and sex on morphometric measurements in pigs is scanty to draw accurate conclusions. Hence, further studies with large sample size are required to draw valid conclusions.

## Authors’ Contributions

PGV, BPK planned and designed the whole study. BVS assisted in sample collection and performing the experiment. BPK, BE, MGP and KSR helped during manuscript writing, cross checking, and revision. BPK and MGP played a helping hand role in data analysis. All the authors read the manuscript and approved the final manuscript.
